# Supplementary material for: SCD2 Regulation Targeted by miR-200c-3p on Lipogenesis Alleviates Mesenchymal Stromal Cell Senescence
Source: Int J Mol Sci. 2024 Aug 5;25(15):8538. doi: 10.3390/ijms25158538 (PMC11313047; doi:10.3390/ijms25158538)
Supplement: Supplementary file 1 [file ijms-25-08538-s001.zip › Table S1.pdf]

Ethical Review Form for Experimental Animals, School of Basic Medical Sciences, Jilin University, China

Approval No. 202027

<table>
<tr><td colspan="7">Name of experiment：SCD2 Regulation Targeted by miR-200c-3p on Lipogenesis Alleviates Mesenchymal Stem Cell Senescence</td></tr>
<tr><td colspan="7">Project source: National Natural Science Foundation of China (NSFC) top-level project</td></tr>
<tr><td colspan="7">Aim: To investigate the role of SCD2 in the regulation of mesenchymal stem cell senescence through influencing lipid metabolism</td></tr>
<tr><td colspan="7">Laboratory Animal Use License Number： SYXK 2018-0001</td></tr>
<tr><td>Application for use Laboratory Category</td><td colspan="6">☐ General environmental facilities √ Barrier Environment Facility
☐ Animal Biosafety Level II Laboratory</td></tr>
<tr><td>Project Leader
Xu He</td><td>Unit</td><td colspan="2">College of Basic Medical Sciences</td><td>Contact Number</td><td colspan="2">0431-85619481</td></tr>
<tr><td>Project applicants
Xu He</td><td>Unit</td><td colspan="2">College of Basic Medical Sciences</td><td>Contact Number</td><td colspan="2">0431-85619481</td></tr>
<tr><td rowspan="5">Proposed use of animals</td><td colspan="3">Animal source: Changchun Yise Laboratory Animal Technology Co.</td><td>Animal breeds, strains</td><td colspan="2">Wistar rat</td></tr>
<tr><td>Animal Grade</td><td>SPF</td><td>Norm</td><td>1-2 months of age, male</td><td>Quantity</td><td>60 pieces</td></tr>
<tr><td>Anesthesia route:</td><td colspan="2">None</td><td>Name of anesthetic：</td><td colspan="2">None</td></tr>
<tr><td colspan="2">Method of execution: euthanized by carbon dioxide inhalation</td><td colspan="4">Disposal of animal residues：placed in a designated freezer at the end of the experiment and disposed by a medical waste disposal company.</td></tr>
<tr><td>Emplacement date</td><td colspan="2">2021.1.1</td><td>结束日期</td><td colspan="2">2024.9.30</td></tr>
</table>

Overview of the content of animal experiments:

This experiment was conformed to the requirements of experimental animal ethics and welfare, and was carried out in the barrier environment of the Animal Experiment Center of the School of Basic Medical Sciences of Jilin University under the supervision of the Committee of Experimental Animal Ethics and Welfare of the School of Basic Medical Sciences of Jilin University. The feeding conditions were in strict accordance with GB14925-2010, and the experimental animals were free to drink and feed.

1. Rats were euthanized by carbon dioxide inhalation, followed by immersing in 75% ethanol for disinfection of the whole body. Then MSCs were isolated from the bone marrow of limbs and cultured using the whole bone marrow adherent method.

2. MSCs were cultivated about 10 generations through *in vitro* serial passages. Then the cellular senescence in different passages was identified, and the replicative senescence model of MSCs was then established. P2-P3MSCs were used as early passage MSCs (EPMSCs) and P9-P10MSCs were used as late passage MSCs (LPMSCs).

3. Rat feeding was managed according to the requirements of the barrier environment facility, and kept in the barrier environment facility with 24h free diet. Animal feed, bedding and drinking water were sterilized and disinfected, and the cages and bedding were cleaned and replaced regularly. The animal residues were placed in the designated freezers at the end of the experiments, and were disposed by the Medical Waste Disposal Company.

Declaration: I will consciously accept the supervision and inspection by the Laboratory Animal Ethics Committee of College of Basic Medical Sciences, Jilin University, and guarantee the objectivity and reliability of the above materials.

Signature of the project leader: Xu He

Signature of the project executor: Xu He

**Basis of review**	1. Whether the project must use animals to conduct experiments? i.e. whether non-living methods such as computer simulation, cell culture, etc. can be used to replace animals? Whether lower animals can be used to replace higher animals for experiments?<br>2. Whether the strains, quality grades and specifications of the animals used in the experiments are appropriate? And whether the number of animals can be reduced by improving the design scheme or using high-quality animals?<br>3. Whether it is possible to optimize the experimental plan and treat animals well by improving the experimental methods, adjusting the experimental observation indexes, and improving the method of killing animals?
**Review Results**	**Laboratory Animal Ethics Committee Approval Opinion:**<br>Agree<br>Signature of the head member of the Ethics Committee (seal)
